# Developing Scotland’s First Green Health Prescription Pathway: A One-Stop Shop for Nature-Based Intervention Referrals

**DOI:** 10.3389/fpsyg.2022.817803

**Published:** 2022-04-05

**Authors:** Viola Marx, Kimberly R. More

**Affiliations:** ^1^Dundee City Council, Dundee, United Kingdom; ^2^Department of Psychology, University of Dundee, Dundee, United Kingdom

**Keywords:** green health, Green Health Prescription, social prescribing, nature-based intervention, referral pathway, public health, social prescribing/social prescription

## Abstract

**Introduction:**

Lifestyle modifications are part of comprehensive treatment plans to help manage the symptoms of pre-existing chronic conditions. However, behavior change is notoriously difficult as patients often lack the necessary support. The present manuscript outlines the development of a Green Health Prescription pathway that was designed to link patients with appropriate lifestyle interventions (i.e., nature-based interventions) and to support attendance. Strengths, Weaknesses, Opportunities, and Threats (SWOT) analysis was undertaken in three focus groups (i.e., National Health Service healthcare professionals, service-users, and nature-based intervention delivery partners) to highlight areas of strength and weakness within the proposed pathway prior to delivery. The SWOT analyses revealed that the pathway was supported by all three focus groups. Weaknesses and threats were identified including sustainability of nature-based interventions in terms of funding, the need to connect patients with appropriate interventions based on their physical and mental health needs, and the requirement to have a “one-stop shop” for information to ensure that the pathway was accessible for all service-users and healthcare professionals. Results were addressed and considered throughout the development of the pathway.

**Discussion:**

The Green Health Prescription pathway was launched in 2019 and gave patients the ability to receive a prescription from a healthcare professional, community service, or to self-refer. The pathway allows patients to contact a consultant, via a telephone service, who is trained to match them with a programme that the patient believes will be enjoyable and that fits their treatment needs. Data collection to assess the efficacy of the pathway is ongoing.

## Introduction

Worldwide, 60% of deaths are traceable to a chronic illness (World Health Organization, 2005). Such illnesses can place a burden on the healthcare system. For example, patients with chronic illnesses account for approximately 80% of all general practitioner appointments in the United Kingdom where they are twice as likely to be a hospital patient. Although medication can be an important component of a treatment-plan, it has long been recognized that lifestyle modifications can help manage the symptoms of pre-existing chronic conditions including cardiovascular disease (Chow et al., 2010; de Lorgeril and Salen, 2011), type 2 diabetes (Chen et al., 2015), obesity (Biedert, 2003), and mental illnesses (e.g., anxiety and depression; Martinsen, 2008), among others. Unfortunately, lifestyle modifications are not an obvious form of treatment for patients (Jarbøl et al., 2017) and even if prescribed by a physician, patients have difficulty engaging with and reaping the benefits of these lifestyle modifications as support is often unavailable (McAvoy et al., 1999; Svetkey et al., 2009). The present manuscript outlines the policy development of a Green Health Prescription pathway, by Dundee’s Green Health Partnership, that was created with the goal of linking patients to lifestyle nature-based interventions that could be prescribed by their healthcare professional (e.g., general practitioner, nurse, and healthcare specialist), community service, or be accessed through self-referral.

### Evidence for Nature-Based Interventions

Engaging in nature has been linked to improvements in both physical (e.g., normalizing blood pressure) and mental health outcomes (e.g., lower incidence of mental illnesses such as depression and anxiety; Shanahan et al., 2016). Nature-based interventions capitalize on these benefits and use a structured programme designed to engage participants with nature (Cox et al., 2017; Shanahan et al., 2019). For example, exercising in greenspace improves anxiety, depression, and general mood post-activity regardless of exercise type or intensity (e.g., walking, cycling, fishing; Pretty et al., 2007; Barton and Pretty, 2010). Moreover, meta-analytic evidence has shown that engaging in greenspace leads to reduced salivary cortisol, heart rate, high-density lipoprotein cholesterol, diastolic blood pressure, and triglycerides (Twohig-Bennett and Jones, 2018). Preliminary evidence also suggests that exposure to greenspace leads to reduced incidence of stroke, hypertension, coronary heart disease, and asthma. Although accumulating preliminary observational and intervention evidence supports the relationship between exposure to greenspace and enhanced physical and mental wellbeing, making use of the outdoors purely for health objectives does not motivate everyone and for many people, other factors, such as social engagement and support (Ingledew and Markland, 2008), are the driving force behind their use of greenspace activity programs.

### Social Support and Health Engagement

Engaging in healthy behaviors as part of a group has direct benefits to the individual as well as benefits for sustained behavioral engagement. Social support has been shown to directly lead to improved mental wellbeing and improved management of chronic conditions in both correlational (Harandi et al., 2017) and experimental (Brunelli et al., 2016) meta-analytic studies. Social support has also been found to form naturally in group settings without being targeted directly by an intervention. This process occurs through informal social support, which refers to support that is offered by an unpaid person (McCamish-Svensson et al., 1999). For example, social support has been found to occur naturally in exercise classes (Christensen et al., 2006), the workplace (Harris et al., 2007), and in online (Wright, 2002), and in-person community groups (Groh et al., 2008). Further, engagement in lifestyle modifications within a group setting has potential benefits directly on the maintenance of behavior change through social identity modification. Individuals who form social identities around a health behavior or activity within a group setting are more likely to sustain engagement over time (Chatzisarantis et al., 2009; Buckingham et al., 2013).

### Local Need for Green Health Referrals

In Scotland the Our Natural Health Service (2020) policy has been developed by NatureScot, Scotland’s Nature Agency. The policy aims to address mental health issues, physical inactivity, and health inequalities with the goal of tackling public health issues with cross-sectorial action by increasing access to outdoor engagement and contact with nature. As a result, four Green Health Partnerships have been developed implementing ONHS locally. All four Green Health Partnerships take different approaches to applying ONHS.

In Dundee, a large selection of nature-based interventions were available locally delivered by independent third sector organizations, however, only a limited number of those were accessible through Dundee’s Sources of Support programme. The Sources of Support programme connects patients identified by general practitioners with dedicated Link Workers. Link Workers are sources of support staff, embedded within primary care (i.e., first point of contact in the healthcare system that is accessible without a referral such as a General Practitioner or Pharmacy), receiving referrals for patients to access non-medical and community-based support. The focus of the local Sources of Support programme is supporting means of self-management of illness by helping people access non-medical social support and resources within the community targeted at helping individuals with their experienced health problems (Dundee Health and Social Prescribing Partnership, n.d.). This programme predominantly focuses on issues relating to debt and financial inclusion, employment and employability, housing and tenancy support, isolation, family and relationship issues, other psychosocial needs, and lifestyle, including physical activity. However, the Sources of Support programme is limited by the Link Worker’s capacity, due to the intensive nature of providing direct support to patients, and thus allows only a selected number of identified patients within primary care to benefit from participating in nature-based interventions. Additionally, although nature-based interventions are part of lifestyle and physical activity, they are not at the forefront of the Sources of Support service and included activities were limited, not covering the entire local service provision.

In the past, several independent secondary care services (i.e., specialist healthcare services that are available by referral from a primary care provider, such as the Clinical Psychology Team) made attempts to create an overview of available nature-based interventions for referral purposes, however, this task was unsustainable due to its time demands for keeping the service provision current. In Dundee, nature-based interventions are predominantly available through several independent third sector partners, providing a rather dynamic environment wherein locations, timings, and type of activity can quickly adapt to the community’s needs, which makes it difficult for a potential referrer (i.e., link worker, general practitioner, nurses, and specialists) to stay informed about the current service provision.

The current provision limitations highlight the opportunity for a more efficient referral pathway to nature-based interventions to improve patient wellbeing and to reduce health inequalities among individuals with chronic health issues. Although access to nature-based interventions and greenspace might not seem relevant to helping with issues related to health inequalities, preliminary research has shown that socio-economic inequalities can be improved significantly by accessing greenspace (Mitchell and Popham, 2008; Hartig et al., 2014; Gascon et al., 2015), and that access is related to reduced acute-stress (Hartig et al., 2014), which enables people to manage ongoing stressors more efficiently, and leads to improved overall self-rated health and wellbeing (Mitchell and Popham, 2007; van den Berg et al., 2015).

The present project aims to explore whether developing a formal referral pathway to nature-based interventions for patients with an identified health need is feasible, through developing a green health referral pathway – Green Health Prescriptions. To our knowledge a formal referral pathway allowing healthcare providers holistic access, regardless of whether they are situated in primary or secondary care, to direct patients to community-based nature-based interventions, was non-existent in the United Kingdom and internationally at the time of data collection. Thus, this manuscript explores (1) whether there is a need for a formal referral pathway informed by different stakeholders (potential service-users, healthcare professionals, and nature-based intervention deliverers), (2) how this referral pathway should be implemented and governed, and (3) potential implementation barriers.

## Methods

### Stakeholder Focus Groups

To address the identified service gap and lack of pathway between nature-based interventions, healthcare professionals, and potential service users, three stakeholder focus groups were held (NHS Tayside healthcare professionals, nature-based intervention deliverers, and potential service-users). The stakeholder groups were designed to be representative of the target population of the proposed pathway including those who would administer the prescription (i.e., NHS Tayside healthcare professionals such as general practitioner (GPs), nurses, specialist nurses, Allied Healthcare Professional lead, public health promotion staff, health intelligence officers, lead pharmacists, and specialty consultants), those who would receive the prescription (i.e., potential service-users including carers, older people with chronic illnesses, and adolescents, young adults and adults with a history of mental health issues) and those who would administer the nature-based intervention (i.e., health walk leaders and coordinators, community garden facilitators, Leisure Trust activity coordinators, MacMillan Cancer Support Coordinators and activity leaders, community trust football club activity coordinators and leads, local third sector interface representatives, members of the Dundee Volunteer and Voluntary Action community and a Sources of Support Link Worker).

### Materials

All groups received a 10-min presentation on evidence-based green health benefits, breadth of local nature-based intervention activities, and the potential to co-design a direct pathway to access those via the healthcare system and other local support services. All groups were asked whether the development of a referral pathway to nature-based interventions is of interest and with that separate Strengths, Weaknesses, Opportunities, and Threats (SWOT) analyses conducted for each focus group, discussing the potential development of Green Health Prescriptions in Dundee, Scotland. Specifically, participants were asked what “strengths,” “weaknesses,” “opportunities,” and “threats” they perceived for the proposed Green Health Referral pathway (i.e., a pathway that linked patients to all local service provision of nature-based interventions through healthcare referrals). SWOT analysis is a tool used to design a product using perspectives and input from various types of stakeholders to better understand how strengths can be capitalized upon, opportunities can be ceased, and how threats and weaknesses can be minimized in the product design period (e.g., Kotler, 2000).

### Participants

#### NHS Health Professionals

The NHS focus group (*n* = 25) discussed the use of nature-based interventions for patient’s prophylactic, treatment, and recovery. Participants were asked in advance to prepare answers to the following questions: (1) “Do you currently ask people/patients if they do purposeful physical activity (just like you might ask if they smoke),” (2) “Are there any aspects of that conversation with the patient that you would be uncertain about?,” (3) “Do you think there would be issues with offering some people/patients Green Health Prescriptions?,” (4) “If you currently promote opportunities to people/patients that encourages them to do activities outdoors what help do you need to do more of that?,” and (5) “Would you like the opportunity to shadow a programme that is currently delivered in the outdoor environment to get a better understanding of how it all works?”

#### Service-Users

A community engagement event (i.e., focus group, *n* = 10) was arranged through Dundee Voluntary Action, which is now Dundee Volunteer and Voluntary Action (DVVA). DVVA is an independent charity ensuring that the third sector is resilient and robustly delivering high quality services for Dundee. At the event, members of the community were asked to discuss how they currently receive information, how they would like to receive information about available services in the future, if they would like to make use of local nature-based interventions, and how green health could be made most accessible to them.

#### Nature-Based Intervention Delivery Partners

Local activities happening outdoors were sought by contacting the third sector interface, by performing Google and Facebook searches, as well as by asking identified nature-based interventions about other interventions they know of. Following sourcing of potential delivery partners, nature-based intervention representatives were invited to discuss the need, development, and potential of a Green Health Prescription pathway in Dundee, Scotland (*n* = 18). This included partners from Leisure and Culture Dundee, Dundee City Council, and local third sector organizations (e.g., health walks, community gardening, community football, and cancer groups) including community groups and friends of parks groups (volunteers which have a vital role in enhancing local parks, leading on projects responding to community needs, and running events).

### Procedure

All stakeholder groups discussed SWOT to the development of a dedicated pathway to access nature-based interventions. SWOT analysis is a qualitative analytic technique that takes a holistic approach by assessing a product from various viewpoints using various focus groups to help avoid biases (Ying, 2010). This technique is valuable when designing a new product, but is not meant as a means of formal product assessment in terms of efficacy. A SWOT analysis was conducted with each group, which were subsequently used to guide the development of the Green Health Prescription pathway.

All stakeholder focus groups were held in person and chaired by NHS Tayside Directorate of Public Health, Senior Health Promotion Officer, and Dundee City Council Greenspace Team Leader. The NHS Tayside healthcare professionals met at Kings Cross in a boardroom. Participants sat in a U-shaped table formation facing each other, with a white board at the front so that it was visible by all members. The whiteboard was used to collate responses from the SWOT throughout the meeting.

Following an introduction session with the attendees, Dundee’s Green Health Partnership Coordinator presented the Green Health Partnership proposal and the vision on creating a Green Health Prescription pathway for NHS healthcare professionals (e.g., general practitioners, nurses, and healthcare specialists) to link patients to nature-based interventions.

The service-user focus group was arranged through DVVA and took place at DVVA’s head office, in a meeting room and were sat around a large round table facing to the front of the room, where the whiteboard was used to populate the SWOT. The session was introduced by DVVA’s Older People Service Manager. Participants introduced themselves and their experience of engaging with services, particularly around mental health, and social care. All participants had a history of accessing community and statutory support services and were identified as having mental and/or physical health issues.

Nature-based deliverer leads met in a seminar room at Abertay University. All participants were seated at chairs with attached tables which were moveable. Participants sat in a U-Formation facing the whiteboard at the front.

## Results

Dundee’s Green Health Prescription pathway was developed based on the focus group discussions and subsequent SWOT analysis. The results from the analysis for each focus group are discussed below.

### NHS Health Professionals

#### Strengths

An identified need was established for primary and secondary care to have direct access to a broad spectrum of nature-based interventions, to ensure that, by the means of a simple referral pathway, more patients will be able to benefit from participating in such interventions. Several strengths of creating a Green Health Prescription pathway in collaboration with DVVA’s Dial-OP and GO projects were identified. Dial-OP and GO is an information hotline open to the public and professionals offering information about local support services and activities. Additionally, it provides a free outreach service to connect vulnerable and isolated people and offers appointment reminders free of charge. A large offering of local nature-based interventions available to the community was established as a strength as well as the ability to tailor to the needs of individual patients through external green health telephone consultation (i.e., a person-centered approach) via Dial-OP and GO. Through the existing information hotline, person-centered consultation, and communication with nature-based interventions to ensure that the service directory is up to date would be possible. Additionally, existing volunteer buddies are available through DVVA’s GO Project and can help patients access nature-based interventions initially.

Another identified strength was linking in with existing pathways and potentially having healthcare professionals accompany patients when already part of their treatment, depending on the service. Lastly, at the time of the consultation primary care was undergoing redesign and the Sources of Support services programme was in the process of expanding, which provided an opportunity to integrate green health prescribing within new service plans.

#### Weaknesses

Weaknesses to engaging with nature-based interventions were discussed. Identified barriers included a lack of knowledge of existing nature-based interventions by healthcare professionals, gaps in service provision for specific groups (e.g., teenagers with brain damage), the potential of patients not taking up referrals and specific uncertainty concerning whether patients would accept nature-based interventions as a viable treatment option, a need to engage with Scottish Index of Multiple Deprivation (SIMD) cluster level 1 (i.e., the most deprived), travel costs for patients to activities, failure to develop digital support tools for patients, joining up other physical activity referral routes in Dundee to avoid duplication across pathways, and concern that nature-based interventions are only funded short-term. These weaknesses were at least partially based on the notion that previous attempts to refer patients to nature-based interventions were only partially successful. Specifically, the clinical psychology team had previously recruited a volunteer to collate nature-based intervention information and other third sector activity offerings, but these lists frequently became outdated, and the system was not sustainable in the long-term. Considering this, confidence in service provision was identified as being critically important to the sustainability of the pathway.

#### Opportunities

Opportunities of the green-health prescription service provision were identified and included further links with allied health professionals, primary and secondary care benefiting from access to Green-Health Prescriptions, and patients gaining transferrable skills and becoming volunteers within a nature-based intervention programme. Additionally, participation in nature-based interventions could lead to further progression of existing groups (e.g., Friends of Parks Groups) and result in employment opportunities for the public; Green Health GO volunteers could be trained to support patients with specific support needs, and nature-based interventions could be linked in with a new local information system for Scotland website (ALISS) to raise profiles of nature-based interventions and increase self-referrals.

Another identified opportunity was that NHS Tayside healthcare professionals have the option to help choose activities for patients, but do not have to as Dial-OP allows for person-centered consultations and the potential to embed a consented patient progress feedback for the prescriber was mentioned. With DVVA’s ongoing support there is an opportunity to create sustainable behavioral change through the engagement with nature-based interventions for patients. It also provides an opportunity for prescribers to try nature-based interventions prior to referring patients. By working in partnership there is an opportunity for collaboration with local partners for joining up existing referral pathways and exploring travel support for patients, which is often identified as a barrier. Having a coordinated network would allow for quality assurance and support outreach (e.g., Green Health Prescribing provides an opportunity for people in need to access the service without seeing a health professional via self-referral).

#### Threats

Lastly, potential threats were identified and included the need for a green health quality assurance to ensure that the projects patients are referred to are safe and meet health and safety standards. Concerns regarding whether there were enough volunteers to successfully run the pathway were also raised. Another identified threat was the potential of creating dependencies as some vulnerable patients easily become dependent and form attachments to certain coaches. Finally, the nature-based intervention capacity was deemed to be a threat if demand for these programmes grew too quickly as community gardens are under-resourced to provide dedicated staff time to deliver further opportunities for the Green Health Prescription pathway.

All results can be seen in detail in Table 1.

**TABLE 1:** NHS stakeholder strengths, weaknesses, opportunities, and threats (SWOT) analysis.

Strengths	Weaknesses	Opportunities	Threats
Identified need for both primary and secondary care for a simple referral pathway to a broad spectrum of NBIs, allowing more patients to benefit by participating	Previously, individual clinical services collated third sector activities via volunteers, but activity lists became outdated quickly. The system was not sustainable in the long-term. This issue needed to be avoided in the new pathway	Available to all patient-facing NHS staff. Connect with Allied Health Professionals, Pharmacists, Specialist Nurse Practitioners, support workers, primary and secondary care to access Green-Health Prescriptions	Project quality assurance required to ensure groups are safe and meet health and safety standards
Existing third sector-based information hotline can be linked in with to create the Green Health Prescription pathway rather than designing a service from scratch	Transport costs for patients to activities and patient’s willingness to fund these transportation costs	Transferrable skills for participants through volunteering within a NBI programme, leading to employment opportunities within the community	Delivery capacity was deemed to be a threat if demand for these programmes grew too quickly
A large offering of local NBIs that were already available to the community was established	Uncertainty as to whether patients would accept NBIs as a viable treatment option	Opportunity for prescribers to try NBIs prior to referring patients	Volunteers need to be trained to handle complex health needs
Ability to tailor to the needs of individual patients through external green health telephone consultation (i.e., a person-centered approach) via pre-existing information hotline by DVVA	Gaps in existing service provision for specific groups (e.g., teenagers with brain damage)	Create a person-centered pathway, where the individual chooses an activity suitable to their needs and to their enjoyment	Potential of creating dependencies as some vulnerable patients can become dependent and form attachments to certain coaches
Existing volunteer buddies are available through DVVA’s GO (Buddy) Project and can help patients access NBIs initially as a means of social support	Possible duplication among existing physical activity referral routes. Central point of information needed to avoid duplication and connect systems	NBIs could be linked in with a new local information system for Scotland website to raise profiles of NBIs and increase self-referrals	
Linking with existing referral pathways and opportunity for healthcare professionals to accompany patients as part of their treatment, depending on the service	Sustainability was raised as a concern if the NBIs are only funded short-term	Healthcare professionals could help choose best options for patients, but do not have to as Dial-OP allows for person-centered consultations	
At the time of the consultation primary care was undergoing redesign and the sources of support services programme (social prescribing) was in the process of expanding, which provides an opportunity to integrate green health prescribing	Previous attempts to refer patients to NBIs were only partially successful as patient initial engagement was low and diminished over time	Opportunity for collaboration with local partners for joining up existing referral pathways and exploring travel support for patients, which is often identified as a barrier	
	Healthcare professionals unaware of existing NBIs delivered by the third sector	Embed consented patient progress updates for the prescriber upon request. Novel compared to existing programmes	
		There is an opportunity to create sustainable behavioral change through the engagement with NBIs for patients	
		Opening the pathway to self-referrals	

### Service-Users

#### Strengths

Several strengths were identified by service-users, including an easy to access “one-stop shop” for information compared to traditional pathways that include the distribution of several leaflets, which patients must go through themselves to find relevant information. Service-users also liked the idea of being able to pick up the phone and speak to someone, without having to meet face-to-face, as it can be difficult to leave the house (physically) or there may be a lack of time due to other responsibilities and time barriers (i.e., caring responsibilities, and employment).

#### Weaknesses

Service-users identified several weaknesses including the concern that it might be difficult to integrate a new pathway successfully within all services where patients are looking for help. Specifically, it was highlighted that it could take a significant amount of time and effort to bring together all the services needed to be accessible through one point of contact.

#### Opportunities

There was a perceived opportunity to have quick access to information through a dedicated worker and thereby omitting the need to go through several leaflets. Many patients reflected on not being capable of searching for the information due to limited time (with barriers to time being other duties such as caring), and the information – as currently delivered – was deemed inaccessible as leaflets are too wordy, lengthy, and repetitive, and thus are often thrown away as finding the key information is too time consuming. Service-users recognized an opportunity to create various contact options (e.g., phone conversation, text message, and email). By making use of DVVA’s established Dial-OP and GO service there is an opportunity to integrate a new pathway within existing service provisions making it easier and more recognizable for service users to engage with.

#### Threats

Service-users identified threats to the Green Health Prescription pathway, such as reliance on leaflets. Service-users reflected on how the new pathway needed to be simple enough to direct people to the source of information without the need to read through a brochure. That is, the pathway needed to create something that is more engaging and taken more seriously than a leaflet.

All results can be seen in detail in Table 2.

**TABLE 2:** Service user SWOT analysis.

Strengths	Weaknesses	Opportunities	Threats
Create an easy to access “one-stop shop” for information compared to traditional pathways that include leaflet distribution, where patients have to find relevant information independently	Difficult to integrate a new pathway successfully within all services where patients are looking for help	Service-users recognized an opportunity to create various contact options (e.g., phone conversation, text message, and email)	Over reliance on leaflets was a perceived threat. The new pathway needs to be simple enough to direct people to the source of information without the need to read through a brochure
Service-users liked the idea of being able to pick up the phone and speak to someone without having to meet face-to-face, as it can be difficult to leave the house (physically) or a lack of time due to other responsibilities (i.e., caring responsibilities/employment)	It could take a significant amount of time and effort to bring together all of the activities to be accessible through one point of contact	By making use of DVVA’s established third sector service (Dial-OP & GO) there is an opportunity to integrate a new pathway within existing service provisions making it easier and more recognizable for service users to engage with	New pathway needs to be more engaging and taken more seriously than a leaflet by users
		Quick access to information through a project worker omits the need to go through leaflets. Leaflets were deemed inaccessible (too wordy, lengthy, and repetitive), and were reported as often being thrown away as finding the key information is perceived as too time consuming	

### Nature-Based Intervention Delivery Partners

#### Strengths

Several strengths were identified by delivery partners, including the belief that patient participation in nature-based interventions could increase confidence to ensure that they eventually feel confident in joining mainstream groups (e.g., jogging clubs). Also, participating in nature-based interventions could affect long-term behavior change and have long-term benefits for physical and mental health (i.e., through lifestyle change). Additionally, the pathway provides the possibility for participants to become volunteers themselves, which increases the trained volunteer force. Delivery partners also identified that green health engagement is an evidence-based practice, that nature-based intervention or green health pathway engagement is good for recovery and value for money (“free participation for patients,” as groups get their own funding source), and that personal success stories are relatable and empowering (role models).

Delivery partners identified that by cooperating with existing partnerships, it would be possible to pull together a large variety of offerings, which has the benefit of targeting different patient groups. Additionally, some existing nature-based interventions practice in greenspaces where services from within the hospital have started to hold clinics outside. This allows for conversations to happen out of traditional clinic settings and might enable engagement and increase health benefits, which can be expanded to other existing services within the hospital.

#### Weaknesses

Several weaknesses were identified by delivery partners including a lack of communication between the health sector and nature-based intervention deliverer groups and that the database would need to be kept up to date so that referrers have confidence that the activity is credible. Additionally, access to spaces can be difficult due to lack of signposting, and with an increase in demand for nature-based interventions providers might struggle with capacity to deliver (e.g., the programme “Branching Out” can only deliver three sessions a year with 12 participants). Another identified weakness was that most activities currently offered occur on weekdays only and some initiatives are short term (i.e., 12-week programmes), which leads to concerns of continued engagement after the programme ends. Additionally, delivery partners highlighted that it is difficult to reach isolated individuals (some people who would benefit the most are isolated and do not reach out, which makes it difficult to include them), and that access to resources may be limited (i.e., logistics and availability of equipment).

#### Opportunities

Several opportunities were identified by delivery partners including the current primary healthcare improvement plan changes suggesting that it is a good time for change (Prudom et al., 2018). Scotland-wide there are various funding sources available, which allow for opportunities for joint applications with partners to match funding (i.e., where the applicant puts forward part of the required funding). Working in partnership allows a joint approach with different sectors and the development of new projects, to refer service users between groups, or move existing programmes outdoors. NHS Tayside has their own volunteers and there is the opportunity to build on that connection to support the programme. When working with partners there is an opportunity to ensure that activities bring value beyond the health element for participants (i.e., confidence building for new participants, learning new skills, or working toward the John Muir Award; John Muir Trust, n.d.). By working with Dial-OP and GO it is possible to make use of existing services such as information sharing or reminders for classes through Morning Call.

#### Threats

Several threats were identified including a lack of drinking water and toilet facilities in parks. Additionally, there was no NHS referral pathway to green health in place (i.e., booking appointments, criteria for referral, follow-up, etc.) as NHS Tayside focusses on Tayside-wide projects whereas the Dundee Green Health Prescription can only focus on Dundee-based projects. To that end, prescribers need to be confident in project governance to engage. Specifically, there needs to be a high enough standard to affect change, and the new pathway needs to support existing initiatives and avoid existing staff workload increases as they are already spread too thinly. Additionally, patients need to be matched to services suitable to their condition, needs, and capabilities. Importantly, it was highlighted that this must be an activity that the patient enjoys creating sustainable behavior rather than patients being sent to an activity via a top-down approach. Further threats included concerns that transport and travel to venues can be difficult as many participants do not have access to a car and that nature-based interventions have a finite capacity, which may lead to a lack of resources when demand increases.

All results can be seen in detail in Table 3.

**TABLE 3:** NBI Stakeholder SWOT analysis.

Strengths	Weaknesses	Opportunities	Threats
Participation in NBIs could increase confidence to help joining mainstream groups (e.g., jogging clubs)	Lack of communication between the health sector and NBIs. New database would need to be kept up to date so that referrers have confidence that the activity is credible	Existing NHS services moved clinics outside, for consultations to happen out of traditional clinic settings to increase patient engagement and increase health benefits. There is an opportunity to apply this model to other services	Prescribers need to be confident in project governance to engage. High standard to affect change, and the new pathway needs to support existing initiatives and avoid existing staff workload increases as they are already spread too thinly
NBIs could affect long-term behavior change and have long-term benefits for physical and mental health (i.e., through lifestyle change)	Location access can be difficult due to lack of signposting	Current primary healthcare improvement plan changes provide an opportunity for change^33^	NHS Tayside focusses on Tayside-wide projects whereas the Dundee Green Health Partnership can only focus on Dundee-based projects
NBIs provide the possibility for participants to become volunteers themselves, which increases the trained volunteer force	Potential difficulties in delivery capacity as demand for NBIs increases	Scotland-wide funding sources available, potential for joint application opportunities with partners to access match funding	Lack of drinking water and toilet facilities in parks
NBI engagement is an evidence-based practice, good value for money (“free participation for patients”, as groups get their own funding source)	Majority of activities offered during weekdays and some initiatives are short term (i.e., 12-week programmes), which leads to concerns of continued engagement after the programme end	Partnership working allows a cross-sector joint approach, new project developments, to refer service users between groups, or move existing programmes outdoors	Patients need to be matched to services suitable to their condition, needs, and capabilities
Personal success stories are relatable and empowering (role models)	Difficult to reach isolated individuals (who would benefit the most and do not reach out, which makes it difficult to include them)	NHS Tayside has their own volunteers and there is the opportunity to build on that connection to support the programme	To create sustainable change enjoyable NBIs need to be selected rather than sending patients to an NBI via a top-down approach
NBI partnership working allows a large service provision, which has the benefit of targeting different patient groups	Access to resources may be limited (i.e., logistics and availability of equipment)	NBIs bring value beyond the health element for participants (i.e., confidence building, learning new skills, or working toward the John Muir Award^34^)	Transport/travel to venues can be difficult as many participants do not have access to a car
		Possibility to connect existing services, i.e., welfare calls or reminders for classes through DVVA’s “Morning Call” to increase support	NBIs have a finite capacity, which may lead to a lack of resources when demand increases

## Discussion

### Green Health Referral Pathway Design

Results from the SWOT analyses guided the Green Health Prescription pathway design to address barriers and threats while also making use of strengths and opportunities identified by stakeholders. Specifically, the SWOT analysis results were vital in shaping and designing the Green Health Prescriptions, to ensure that this new referral pathway would become a valuable tool, which is easily used across all health services, well received by the patients, and nature-based intervention deliverers to allow for a smooth transition between services. For example, the highlighted concerns raised by healthcare professionals about the credibility of community-led nature-based interventions was addressed through the creation of a green health quality assessment for nature-based interventions, where all groups needed to have at least two years of future funding, trained staff and volunteers in place with experience in service delivery addressing the stakeholders concern for delivery stability and quality. Suitable nature-based interventions were categorized for suitable patient types (e.g., chair-based exercise classes for patients with mobility issues) addressing stakeholders concern for programs that cater to patient’s physical and mental health needs. The creation of a governed nature-based intervention database was undertaken and connected with the already established information hotline Dial-OP and GO. This task was vital to overcome barriers expressed by healthcare professionals, create confidence in the service provision, to overcome barriers relating to nature-based intervention availability and opening hours (i.e., burden of having to locate information), as well as information relating to service-users activity preferences.

### Establishing a Green Health Directory

To enable referrals across all local nature-based interventions a Green Health Directory was established. Following a scoping exercise of all available third sector green health activities, the *Green Health Directory* was established. This directory holds information about the type of activity available, the kind of suitable service users, programme contact information, which type of support is available, and what kind of funding streams the service uses.

Dundee’s Green Health Directory includes the following activities: health walks, community gardening, nature conservation, antenatal and family walks, running, gentle cycling using eBikes and eTrikes, yoga, tai chi, arts and crafts, walking football, still game, as well as local Friends of Parks groups and are delivered by a variety of established third sector service providers. Additionally, ParkLives are included in the directory with a changing offering depending on demand, but can include activities such as tai chi, Nordic walking, couch to 5 k, kettlercise, Tabata, boxing fitness, circuits, messy play, additional support needs Boccia, and many more. All services, apart from walking football, are free, which is essential for overcoming financial barriers to access for service users.

### Suitability of Nature-Based Intervention Participants

Green Health Prescriptions are able to support the following groups based on a green, amber and red (non-suitable) system: (1) Green: recovery from poor but stable mental health, addiction, or surgery; approaching low mood, people needing walking aids, socially isolated/lonely, cardiovascular disease, cancer, obesity, type 2 diabetes, COPD, asthma, or chronic pain, (2) Amber: (may need accompanied by a carer) dementia, Alzheimer’s, severe autism, severe mobility issues, or vision impairments, and (3) Red: house-bound, unable to travel independently to the activity, unable to participate independently and do not have a care-giver to access activities, or unable to walk far enough to participate.

### Design of Green Health Prescriptions

**FIGURE 1:**
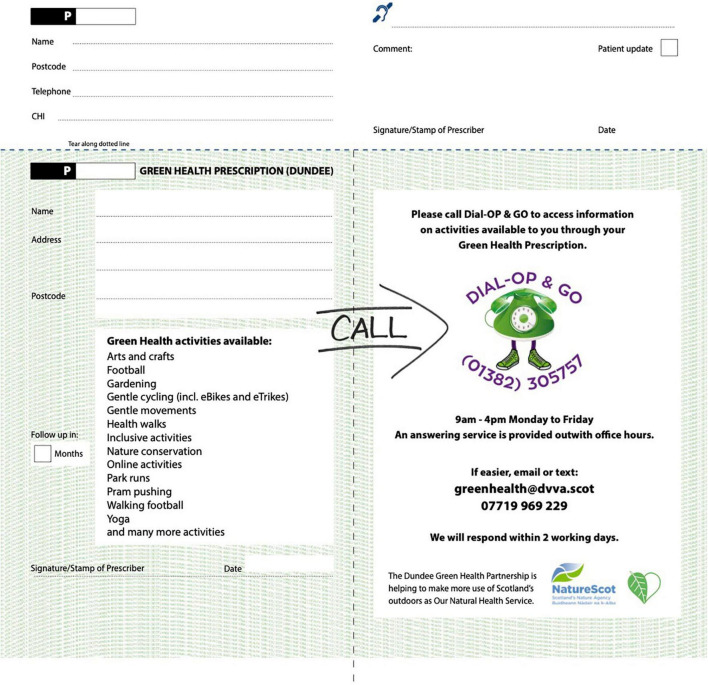
Green Health Prescription front side. © Copyright of Green Health Partnership.

**FIGURE 2:**
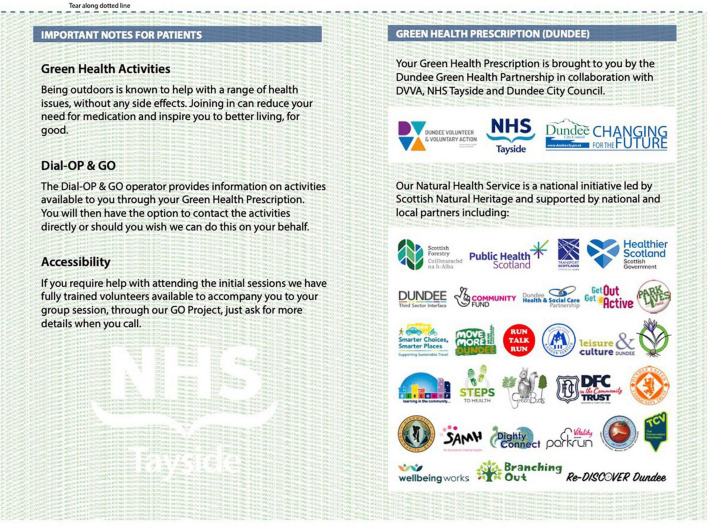
Green Health Prescription back side. © Copyright of Green Health Partnership.

Green Health Prescriptions are designed to match the look and feel of the red colored drug-based prescription available in Scotland, with the Green Health Prescription being green instead. The Green Health Prescription comes in two parts: (1) the main patient facing prescription part, modeled on the drug-based prescription and (2) a narrow admin tear-off slip (see Figures 1, 2). The patient facing section includes patient name, address, breakdown of offered activities, contact information for DIAL-OP and GO Service (phone number, email, text, opening times), prescriber follow-up timeframe, prescriber ID, signature/stamp, and date, and (2) the admin slip includes information about the referral (prescriber ID, patient name, contact number, postcode, CHI (i.e., a unique identifying health code), comments section, communication aid, date, prescriber requested patient update, prescriber signature). Green Health Prescriptions are printed at the NHS national contracted printer where Scottish drug-based prescriptions are also printed to enhance the feel and look of the prescription and referral experience.

### How Does It Work?

After a Green Health Prescription has been issued the patient calls the DVVA’s Dial-OP and GO Information Line service. If the patient does not get in touch, the service will contact the patient within 7 days. A dedicated, full-time Green Health Development Worker will then have a consultation with the patient to find a suitable nature-based intervention within the Green Health Directory and identify further support needed to help the patient engage in the service, such as the DVVA’s Green Health Buddies, through the GO Project. Green Health Buddies can help the service user to attend an intervention to overcome initial barriers and anxieties related to participating in a new activity alone. The Green Health Buddies will meet the service user prior to the activity and then attend the activity with the service user. Once the service user has become more familiar with the activity the Green Health Buddy will no longer attend. This support is limited to a maximum of three times. The service is not a transport service as the service users are expected to be able to attend the activities on their own.

### Prescribers

To gain implementation approval, in October 2018, the Green Health Partnership put forward a proposal to the Local Medical Sub Committee to introduce the Green Health Prescription pathway pilot for use within primary care. Green Health Prescriptions were successfully launched by Joe Fitzpatrick, Scottish Minister for Public Health, Sport and Wellbeing, alongside Drew Walker, Director of Public Health National Health Scotland Tayside, in April 2019 in three participating surgeries: Taybank Medical Surgery, Whitfield Medical Center, and Lochee Medical Center. The Green Health Prescription implementation was administered in three phases.

### Phase 1

#### General Practitioner Surgeries

Three general practitioner surgeries were chosen for the pilot programme starting in April 2019. The three piloting surgeries are situated within regeneration areas and have practice populations experiencing high levels of health inequalities. Taybank Medical Surgery is adjacent to the award-winning community garden, Tayview; Whitfield Medical Center has many nature-based interventions starting from the community center they are based in, and Lochee Medical Center has strong connections with recovery services with the area offering many nature-based interventions. These surgeries cover a cohort of 11% of Dundee’s population and cover target SIMD areas for tackling health inequalities. Depending on the general practitioner surgery model, prescribers could include general practitioners, healthcare assistants, practice nurses, and clinical managers. Each surgery was tasked with deciding which staff would be most suited to engage with the programme.

### Phase 2

#### Secondary Care

Green Health Prescriptions were issued across other allied health professionals services to establish a referral pathway outside primary care for patients already part of the system starting in June 2019. This included health and clinical psychologists, physiotherapists, occupational therapists, consultants, locality pharmacists, chronic pain clinic, The Center for Brain Injury Rehabilitation, adult weight management service, and mental health services.

#### Community Prescribers

Additional prescribers were identified at Dundee’s Job Center Plus as well as within community groups such as Positive Steps, AMINA – Muslim Women’s Resource Center, Integrated Substance Misuse Service, peer support workers, and integrated cancer journey.

### Phase 3

#### Expanding Secondary Care and General Practitioner Reach

Since the prescriptions launch, the service has expanded into further selected secondary care services such as cardiovascular, rheumatology, physiotherapy, clinical psychology, and general surgery among others. Following a year of running the service, the Green Health Directory held 60 weekly green health activities and included over 200 NHS Tayside prescribers within primary and secondary care.

### Coronavirus Disease COVID-19 Adaptation

Following lockdown, and an increased identified need to link patients with support services, 14 Dundee general practitioner surgeries signed up. This brought the total to 17 GP surgeries and additional secondary care services, which resulted in over 310 NHS Tayside prescribers by October 2020. Additionally, the service was promoted on local television, leading to a surge in self-referrals particularly around mental health support.

COVID-19 has forced the adaptation of services to keep people active. Green Health Prescriptions went digital to enable telephone and video consultation referrals; innovative approaches include online activities for all ages and needs such as home yoga/Pilates/gentle movements classes. These activities were chosen based on a survey of participants’ most desired activities. Some nature-based intervention activities went online, such as virtual health walks, or had to stop due to furloughed staff. This adaptation has helped solve several barriers highlighted by the NHS healthcare professionals focus group, including lack of access to green-spaces due to travel constraints.

Additional green health interventions were created to support existing and new service users, as such Dundee’s Nature Prescription Calendar was created based on NHS Shetland’s Nature Prescription which is an activity-based calendar to encourage people to get outside and physically active to improve their mental health without focusing on being active, but on having fun, a more sustainable way of changing lifestyle behaviors. This self-led activity can be done in one’s own time, alone or with the household, and is COVID-19 friendly.

### Generalizability

Approaches to social prescribing are widely seen as a useful tool for the healthcare sector to integrate within practice to provide person-centered care for physical and mental health, and social wellbeing alongside medical treatments (Husk et al., 2019). The development of a prescription-type pathway, which both patients and healthcare staff are familiar with, has been an intuitive referral method for prescribers participating in the Green Health Prescription pathway. Whilst, this type of pathway has solely been used to promote outdoor activities, it provides opportunity for expansion to include all third sector-based community activity.

The key elements to successful pathway creation include: (1) a solid infrastructure linking the patient from the healthcare professional to a dedicated project staff member, this member of staff can be situated within the healthcare-, the health and social care – or third sector, depending on the local system, (2) a governed activity directory; (3) a mechanism to inform the activity consultant that a referrer issued a prescription and (4) sharing consented reasons for referral with the consultant, (4) a person-centered consultation with the patient, (5) pro-active engagement with the patient within a certain timeframe to improve uptake and engagement, and lastly (6) reconnecting with the patient following participation to discuss activity enjoyment, additional support needed or a different activity.

### Comparison to Other Nature Prescriptions

#### Shetland’s Nature-Prescriptions

GPs in Shetland, United Kingdom, are prescribing nature activities to their patients in the form of a calendar, referred to as *Nature Prescription*. The Nature Prescription was launched in October 2018 and encourages patients to engage with nature by supplying monthly activities to be completed in the patient’s own time (RSPB, 2018). The project is led by National Health Service, publicly funded healthcare system in the UK (NHS) Shetland and RSPB Scotland (Royal Society for the Protection of Birds).

#### Edinburgh’s Adaptation of the Shetland Program

Inspired by NHS Shetland’s Nature Prescription, Edinburgh has launched a pilot nature prescribing programme, a leaflet available through GP surgeries, which directs patients to visit local parks, join a walking group, walk between 0.5 and 3 miles in nature in their own time, and includes nature activities (RSPB, 2020). This is a partnership project between RSPB Scotland and NHS Lothian.

#### Park Rx America

*Park Rx America* is a non-profit organization, enabling health care professionals to prescribe nature during routine consultations. The prescription directs patients to a local park, including the visit’s duration and frequency. During the park visit patients are encouraged to listen to birds chirping, listen to the sound of rivers and streams, and look at trees and leaves (Park Rx America).^1^

#### Comparison of Previous Programs With the Green Health Prescription Pathway

Collectively, other nature prescriptions and green prescribing programs are a self-directed practice relying on a patient’s ability to choose a program that is suitable to their needs and on their own motivation to sustain attendance. The Green Health Prescription pathway in Dundee on the other hand focuses primarily on the connection of patients with nature-based interventions, to make use of local activities and connect participants not just with nature but also socially by connecting patients with groups (Chatzisarantis et al., 2009; Buckingham et al., 2013). This is done using a Green Health Development worker who is able to support the patient in choosing the right program, with the right level of support, and by providing a volunteer “buddy” for initial attendance of the programs if needed to enhance social support (e.g., Brunelli et al., 2016).

To address the self-directed practice of other nature prescription/green prescribing models, the Phase 2 development included the Green Health Development Worker to share local self-directed resources with selected patients struggling to engage with group-based activities due personal barriers such as social anxiety, feeling embarrassed, feeling unfit, time constraints, or travel barriers. Self-directed nature activities included sharing health walks, art trails, a cycle map, Dundee’s version of the nature prescription (Dundee Green Health Partnership, n.d.).

It should be noted that since summer 2020 there has been a resurgence of green prescribing projects across England. The COVID-19 pandemic highlighted the benefits of accessing nature for mental and physical health, resulting in ϵ5.77 million funding (NHS England, 2020) in support of implementing green social prescribing nationally. However, currently results of these are not widely shared. This is a major limitation in terms of the ability to replicate these programs, a limitation that has been highlighted by reviews of green health programs (Rugel, 2015). Our hope is that by sharing the details of the development of the Green Health Prescription pathway and how it functions, that it can be replicated in other research, and be used by other communities more broadly.

### Limitations

Although this work is ground-breaking in terms of connecting patients with local nature-based intervention services formally through their healthcare providers using the same route patients are used to when receiving a drug-based prescription, there are still limitations that must be addressed. First, SWOT analysis can be heavily biased due to the participants who are involved (Coman and Ronen, 2009; Phadermrod et al., 2019). To that end, the results presented may not be inclusive of all strengths, weaknesses, opportunities, and threats to the pathway that would have been perceived by the Dundee population as a whole. Thus, it is likely that other barriers to engagement with the pathway that were not addressed in the program design exist. Importantly, the efficacy of the Green Health Prescription pathway is still being evaluated and it is likely that improvements are needed to enhance uptake and maintenance within Dundee.

The application of a prescription-type pathway is certainly generalizable due to its basis in the medical prescription models, but is reliant upon the governing infrastructure. Secondly, since we are still testing the pathway efficacy within Dundee, we cannot attest to the generalizability of this pathway to other communities which will have varying levels of pre-existing nature-based interventions.

Thirdly, the financial support needed for this type of comprehensive pathway might pose difficulties for generalizable implementation. Communities without these provisions in place, would likely need to secure financial support to integrate this type of comprehensive Green Health Prescription pathway, which may hinder uptake. The Green Health Development Worker position in Dundee needed to support this pathway is an externally funded position for establishing the programme. However, there is an opportunity to expand upon the existing workforces job description, if there is a similar social prescribing position in post already.

Finally, and most generally, while it is known that engagement in physical activity is beneficial for disease management (e.g., Kyu et al., 2016), the benefits of engaging in physical activity in nature are still unclear, although preliminary results are promising. Specifically, inadequate reporting of experimental procedures and an overreliance on cross-sectional data, small sample sizes with homogenous populations, and neglecting to account for confounding variables such as socioeconomic status and green space quality have been highlighted as limitations of the current research on nature-based interventions (Rugel, 2015). Thus, while engaging in physical activity - regardless of location (e.g., indoor versus outdoor, green versus urban) - is beneficial for health, more rigorous research, using diverse well-powered samples, is needed to clarify the unique benefits of physical activity engagement in nature.

## Conclusion

The Green Health Prescription pathway in Dundee has been awarded Scottish Public Service Award’s Colin Mair Award for best policy in practice, Paths for All’s Active Travel Project of the Year Award, and the International Social Prescribing Conference’s Best Nature-Based Social Prescribing Project. The project was commended for the United Kingdom-wide Local Government Chronicles and annual Nature of Scotland Awards in the Public Health category, respectively.

Data on the efficacy of the Green Health Prescription pathway is currently being collected for evaluation including consultation contact rates and subsequent nature-based intervention attendance rates. Preliminary analysis of the efficacy of the pathway has shown consultations and activities are equally accessed by users spanning the range of SIMD groups (Smith et al., 2021), however, prescriptions are issued with a focus on people from more deprived backgrounds to reduce health inequalities. That is, the consultation service is as accessible to those who face a high level of deprivation as it is to those who are of a higher economic status. This is promising as making the initial point of contact is necessary for connecting patients with appropriate nature-based interventions. Future evaluations will need to assess the perceived accessibility of information from the point of view of the service-user and long-term outcomes such as attendance to prescribed programmes.

## Data Availability Statement

The original contributions presented in the study are included in the article, further inquiries can be directed to the corresponding author/s.

## Ethics Statement

The studies involving human participants were reviewed and approved by TASC governance (Tayside Medical and Science Center). Written informed consent for participation was not required for this study in accordance with the national legislation and the institutional requirements.

## Author Contributions

Both authors contributed to the article and approved the submitted version.

## Conflict of Interest

The authors declare that the research was conducted in the absence of any commercial or financial relationships that could be construed as a potential conflict of interest.

## Publisher’s Note

All claims expressed in this article are solely those of the authors and do not necessarily represent those of their affiliated organizations, or those of the publisher, the editors and the reviewers. Any product that may be evaluated in this article, or claim that may be made by its manufacturer, is not guaranteed or endorsed by the publisher.

## Footnotes

1
https://parkrxamerica.org/providers/
